# Intraspecific trait variation across elevation predicts a widespread tree species' climate niche and range limits

**DOI:** 10.1002/ece3.5969

**Published:** 2020-04-17

**Authors:** Michael E. Van Nuland, John B. Vincent, Ian M. Ware, Liam O. Mueller, Shannon L. J. Bayliss, Kendall K. Beals, Jennifer A. Schweitzer, Joseph K. Bailey

**Affiliations:** ^1^ Department of Biology Stanford University Stanford CA USA; ^2^ School of Environmental and Forest Sciences University of Washington Seattle WA USA; ^3^ Institute of Pacific Islands Forestry USDA Forest Service Pacific Southwest Research Station Hilo HI USA; ^4^ Department of Ecology and Evolutionary Biology University of Tennessee Knoxville TN USA

**Keywords:** adaptation, climate range, ecological niche models, elevation gradient, functional traits, intraspecific variation, phenotypic plasticity, quantile regression

## Abstract

Global change is widely altering environmental conditions which makes accurately predicting species range limits across natural landscapes critical for conservation and management decisions. If climate pressures along elevation gradients influence the distribution of phenotypic and genetic variation of plant functional traits, then such trait variation may be informative of the selective mechanisms and adaptations that help define climatic niche limits. Using extensive field surveys along 16 elevation transects and a large common garden experiment, we tested whether functional trait variation could predict the climatic niche of a widespread tree species (*Populus angustifolia*) with a double quantile regression approach. We show that intraspecific variation in plant size, growth, and leaf morphology corresponds with the species' total climate range and certain climatic limits related to temperature and moisture extremes. Moreover, we find evidence of genetic clines and phenotypic plasticity at environmental boundaries, which we use to create geographic predictions of trait variation and maximum values due to climatic constraints across the western US. Overall, our findings show the utility of double quantile regressions for connecting species distributions and climate gradients through trait‐based mechanisms. We highlight how new approaches like ours that incorporate genetic variation in functional traits and their response to climate gradients will lead to a better understanding of plant distributions as well as identifying populations anticipated to be maladapted to future environments.

## Graphical abstract

Accurately forecasting plant species distributions and range limits is a central challenge for understanding climate change effects on the landscape. Here, we extend a recently proposed quantile regression approach using within‐species functional trait variation across elevation gradients to predict a widespread tree species' climate niche. Collectively, these findings point towards the ecological and evolutionary drivers of climate range limits and demonstrate how this approach can improve current niche models by including intraspecific trait‐climate responses.

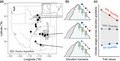

## 1 INTRODUCTION

In a rapidly changing world, a variety of tools and approaches are required to predict species distributions for conservation planning and to achieve management goals. Plant range limits reflect the biotic, energy, and resource constraints that determine where populations are no longer self‐sustaining (Hargreaves, Samis, & Eckert, 2014). Ecological niche models (ENM) are a common method to identify and visualize how environmental factors relate to a species' geographic distribution (Elith & Leathwick, 2009). These approaches typically rely on correlations between climate variables and species occurrences (Araújo & Peterson, 2012; Hijmans, Phillips, Leathwick, & Elith, 2017), but they are not well‐suited to identify the mechanisms explaining how and why species ranges extend across environmental gradients (Dormann et al., 2012). Improving this issue would fill significant gaps in our knowledge of the ecological and evolutionary mechanisms that influence species distributions and their response to environmental change.

Within‐species variation in plant functional traits related to growth and fitness offers an important bridge between correlational models and the ecophysiological drivers of plant‐climate responses. This is because plant trait variation is, in part, a result of the abiotic variables used to parameterize ENM. For example, most ENM for plant species use temperature and precipitation factors that affect the mean and variance of traits among populations and genotypes on the landscape (Barnes, 1975; Clausen, Keck, & Hiesey, 1948; Grady et al., 2011; Mod, Scherrer, Luoto, & Guisan, 2016; Pregitzer, Bailey, & Schweitzer, 2013). Though there are additional sources of “noise” that can influence plant traits (e.g., species' evolutionary history), the expression of functional trait variation at regional and global scales should reflect some aspects of the underlying physical constraints applied by abiotic pressures before biotic interactions mediate phenotypic expression at local scales (Stahl, Reu, & Wirth, 2014). As a result, intraspecific trait variation should be useful for predicting the total extent and limits of a species distribution across environmental gradients.

Double quantile regression was recently used to examine the mechanistic relationship between plant functional traits and climate range limits (Stahl et al., 2014). In general, this approach incorporates functional trait variation across a species' distribution to predict their upper, median, and lower climate extremes, and the resulting response patterns shed light on potential filtering mechanisms and adaptations to climatic range limits. In their original approach, Stahl et al. (2014) used species‐specific trait means for 250 North American tree species, leaving the vast majority of within‐species variation unaccounted for. However, there is a growing recognition that incorporating genetic variation and local adaptation in niche models can improve forecasts of species distributions under current and future climates (Chardon, Pironon, Peterson, & Doak, 2019; Gotelli & Stanton‐Geddes, 2015; Ikeda et al., 2014, 2017; Peterson, Doak, & Morris, 2018; Read, Hoban, Eppinga, Schweitzer, & Bailey, 2016; Valladares et al., 2014). This is clear when acknowledging that population and genetic structure are not homogeneous across tree species ranges (Hampe & Petit, 2005) and geographically variable selection can drive trait adaptations that result in populations with different climatic tolerances (Aitken, Yeaman, Holliday, Wang, & Curtis‐McLane, 2008; Savolainen, Pyhäjärvi, & Knürr, 2007). Similarly, phenotypic plasticity (genetic × environment interactions that result in different trait values under different conditions with the same genotype) should be an important mechanism for plants to cope with environmental change, particularly for long‐lived trees and genotypes near range limits (Gunderson, O'hara, Campion, Walker, & Edwards, 2010; Valladares et al., 2014). Given that genetic variation influences plant ecological and evolutionary responses across environmental gradients (Bailey et al., 2014), it is critical for new approaches to consider within‐species variation to link functional trait mechanisms with species distributions (Fitzpatrick & Keller, 2015; Ikeda et al., 2014; Jump & Penuelas, 2005; Moran, Hartig, & Bell, 2016).

We extend the approach outlined by Stahl et al. (2014) that compared plant trait‐climate relationships across many North American tree species to a large‐scale study of a single dominant, widespread, and genetically diverse tree species in the western US (*Populus angustifolia* James). Specifically, we tested whether intraspecific trait variation could predict the species' climate range, if climate range limits impose constraints on the genetic variation of functional traits, and whether we can use this information to project how trait variation is constrained on the landscape. We surveyed functional trait variation (plant size/growth and leaf morphology) in the field across elevation gradients in distinct watersheds, treating each gradient as a subset of the overall species' phenotypic and climatic range. Then, using a common garden experiment with clonal replicates of *P. angustifolia* cuttings to separate environmental and genetic effects on trait variation, we compared the response patterns in field traits (variation caused by environment and genetic effects) to common garden traits (variation caused by genetic effects). We hypothesized that:

Hypothesis 1 Plant size/growth and leaf morphology traits from field observations predict the species' climate range using double quantile regression

Hypothesis 2 Common garden trait‐climate relationships will show similar response patterns as field traits, indicating the presence of genetic clines that constrain trait variation at range limits.

## 2 METHODS

### 2.1 Species and climate variables

**Figure 1:**
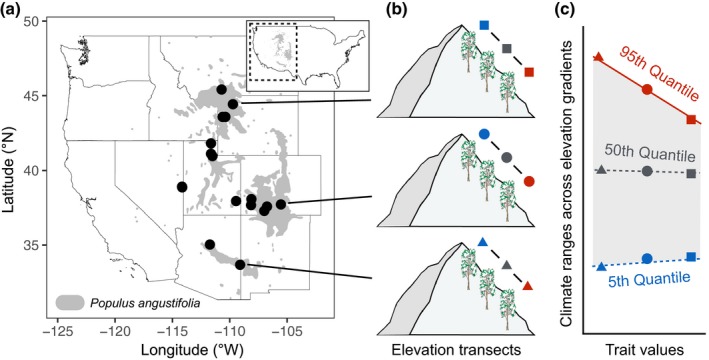
Elevation transects sampling and double quantile regression approach to predict climatic range limits with intraspecific trait variation. We sampled sixteen populations of *P. angustifolia* using elevation transects (Table 1) to capture a broad range of functional trait and climatic variation. We then used double quantile regression to examine how intraspecific trait variation might predict the climate range limits of *P. angustifolia* (adapted from Stahl et al., 2014). Mean functional trait values for trees (field) and clonal cuttings (common garden) were estimated at each transect site and used to predict the upper limits (95th quantile), median (50th quantile), and lower limits (5th quantile) of the species' climate niche. Outer regression lines (blue and red) encompass the overall climate range of the species, and locations outside these lines represent potential “no‐go areas” for the tree species (i.e., areas where no trait value occurs that might allow for that climate habitat to be occupied by *P. angustifolia*). The median regression line (gray) represents the average realized climate niche as predicted by intraspecific trait variation. Solid lines represent slopes significantly different from zero

*Populus angustifolia* James is a dominant riparian species with a wide geographic range throughout the Rocky Mountains (Braatne, Rood, & Heilman, 1996; Little, 1976; Figure 1) and exhibits broad trait variation across the western U.S. (Ernst & Fechner, 1981; Van Nuland, Ware, Bailey, & Schweitzer, 2018; Ware et al., 2019). This makes it an excellent species to examine how dominant climate gradients and intraspecific trait variation relate to the species' ecological niche. We performed a large randomized sampling field survey in June of 2012—with the intention of covering as much variation as possible—in which putative *P. angustifolia* genotypes (total *n* = 557) were sampled and marked with GPS points (Oregon^®^ 550t) (see Field sampling and common garden experiment below). Using the georeferenced sampling points, we gathered 15 climate variables at 30 arcsecond resolution from the Environmental Rasters for Ecological Modeling dataset (ENVIREM; envirem.github.io). These variables encompass annual and monthly trends of temperature and moisture‐related factors are likely to be directly related to the ecophysiological processes that influence species geographic ranges, and in some cases outperform WorldClim variables in ENM (Title and Bemmels, 2017). We used the following ENVIREM variables: climatic moisture index, aridity (Thornthwaite index), annual potential evapotranspiration (PET), continentality, growing degree days >0°C, growing degree days >5°C, maximum temperature of the coldest month, minimum temperate of the warmest month, number of months with mean temperature >10°C, PET of the driest quarter, PET of the wettest quarter, PET of the coldest quarter, PET of the warmest quarter, PET seasonality, and thermicity (global compensated index).

### 2.2 Field sampling and common garden experiment

We sampled natural occurring stands of *P. angustifolia* in sixteen watersheds from southern Arizona to southern Montana (Figure 1). These river systems included: Blue River and Oak Creek, AZ; Medano Creek, Park Creek, San Juan River, Dolores River, and San Miguel River, CO; Lexington Creek and Snake Creek, NV; Indian Creek, Ogden River, Logan River, and Weber River, UT; Snake River and Gros Vente River, WY; and Yellowstone River, MT. The specific sampling approach has been described previously (Van Nuland et al., 2018; Ware et al., 2019). Because populations are typically confined to riparian zones within distinct watersheds (Braatne et al., 1996 **)**, we sampled 5–10 putative genotypes of mature *P. angustifolia* trees from three sites along an elevational transect in each watershed (Figure 1; Table 1) to capture intraspecific variation in traits and climate habitats (Table S1; Figure S1). We measured the diameter (DBH) of individual trees in the field (though *Populus* genets can be multi‐stemmed and these measures may only reflect the size of individual shoots) and collected cuttings from low‐hanging branches (2–2.5 m canopy height) for clonal propagation of putative genotypes in the common garden experiment (see below). Five fully flushed leaves were collected from multiple locations 360° around the tree at the same canopy position (2–2.5 m canopy height). We calculated specific leaf area (SLA; average of five leaves per putative genotype) with scanned measurements of leaf area (WinFolia software; Regent Instruments Inc.) and leaf dry mass (Cornelissen et al., 2003). Although leaf traits respond to gradients of UV radiation and light spectra that naturally vary with altitude and latitude, we are unable to disentangle these effects from climate variables based on our sampling design. Leaf morphology also did not strongly correlate with tree DBH (DBH‐leaf mass: *r* ^2^ = 0.001, *p* = .2; DBH‐leaf area: *r* ^2^ = 0.001, *p* = .04; DBH‐SLA: *r* ^2^ = 0.001, *p* = .5).

**Table 1:** Locations and elevation ranges of *P. angustifolia* transect sites

Population	Latitude	Longitude	Elev. 1	Elev. 2	Elev. 3
Blue River, AZ	33.6677	−109.0931	1,710.1	1,827.8	2,044.4
Oak Creek, AZ	35.1435	−111.6436	1,625.2	1,720.5	1,929.0
Medano Creek, CO	37.7155	−105.5356	2,377.0	2,459.8	2,497.2
Park Creek, CO	37.5748	−106.7000	2,484.4	2,570.0	2,638.2
San Juan River, CO	37.2837	−107.0709	1,855.7	2,020.7	2,726.3
Dolores River, CO	37.6713	−108.1424	2,296.1	2,495.6	2,808.2
San Miguel River, CO	38.0722	−108.1118	1,777.5	2,098.7	2,685.8
Lexington Creek, NV	38.8604	−114.1673	1,892.6	2,133.7	2,321.0
Snake Creek, NV	38.9212	−114.1490	1,712.0	1,876.0	2,337.2
Indian Creek, UT	37.9460	−109.4447	1,742.8	1,882.9	2,225.4
Ogden River, UT	41.1022	−111.6381	1,558.1	1,602.7	2,186.7
Logan River, UT	41.8097	−111.6365	1,529.4	1,686.7	1,859.9
Weber River, UT	40.9566	−111.5084	1,384.0	2,027.5	2,285.8
Snake River, WY	43.5855	−110.6189	1,742.2	1,991.3	2,281.2
Gros Vente River, WY	43.5884	−110.3825	2,052.9	2,144.6	2,244.2
Yellowstone River, MT	45.4210	−110.7124	1,372.9	1,481.8	1,509.4

Note

Latitude and longitude coordinates (degrees) are averaged across all sampling points along an individual transect. Elevation data (meters above sea level) are averages of 5–10 sampling points within a single transect site. Elevation 1 = low, Elevation 2 = mid, and Elevation 3 = high.

We sampled trees at approximately the same aspect that were far apart from each other (>30 m) to try and avoid collecting from the same genet, and subsequent microsatellite analysis was used to identity unique genotypes (see Ware et al., 2019 for specific details). Genomic DNA was extracted from powdered leaf samples (Qiagen DNeasy Plant Mini Kit; Qiagen), multi‐locus genotypes were created from nine microsatellite markers (eight from The International *Populus* Genome Consortium and one from Tuskan et al., 2004), and clone genotypes (trees with a 100% match at all microsatellite loci) were identified in GenAlEx v6.4 (Peakall & Smouse, 2012). Single clones that had inadvertently been sampled more than once comprised <4% of the originally sampled trees, and we kept data from one individual per clone prior to running genetic analyses. Additionally, *P. angustifolia* is known to hybridize with cooccurring Salicaceae species (e.g., *Populus fremontii* and *Populus deltoides*) at lower elevations and form distinct hybrid zones (Floate, Martinsen, & Whitham, 1997; Keim, Paige, Whitham, & Lark, 1989; Whitham, Floate, Martinsen, Driebe, & Keim, 1996). We avoided sampling hybrids by staying at higher elevations (above our visual assessments of hybrid zones, if they occurred) and by not collecting from trees with deltoid‐shaped leaves that are markedly wider with longer petioles than the characteristically lanceolate leaves of *P. angustifolia*.

We created a common garden experiment in June 2012 with clonal replicate cuttings of genotypes from the field to examine the effect of genetic variation on *P. angustifolia* plant traits in relation to the species' climate niche. The specifics of the common garden experiment have also been described previously (Ware et al., 2019). Briefly, multiple branch‐tip cuttings (~20 cm in length) were collected from each putative genotype in the field in June 2012 and grown in an indoor glasshouse at Northern Arizona University. Each cutting was treated with a rooting hormone (indole‐3‐butyric acid [IBA]; Hormodin^®^ 2; OHP Inc.), potted in general potting mix (equal parts peat, vermiculte, and perilite), and randomized in the glasshouse. Cuttings were grown with no supplemental lighting, modest temperature control (within ~5°C compared to outdoor temperatures), and full watering thrice weekly, which allowed them to generally track the normal growing seasons with a dormancy period between November and March. In June of 2013, we measured the diameter of annual growth (i.e., the size of new shoot growth) for plants in the common garden (total *n* = 1,514). In addition, five leaves were collected from multiple locations along the main stem of rooted cuttings for average leaf trait measurements (identical methods as above). We calculated the broad‐sense heritability of these traits as the total amount of variance explained by plant genotype (Connor & Hartl, 2004; Ware et al., 2019) and compared trait variation between field and common garden locations to separate patterns of genetic differentiation from phenotypic plasticity (Connor & Hartl, 2004).

### 2.3 Trait‐climate range response patterns

To test the first hypothesis (that field trait variation will predict climate range limits), we explored relationships between DBH, leaf area, leaf mass, and SLA in the field and climate variables using double quantile regression. These are commonly measured functional traits that impact organismal performance and correspond to the morphological and physiological mechanisms that influence plant fitness and demography (Adler et al., 2014; Cornelissen et al., 2003; Gibert, Gray, Westoby, Wright, & Falster, 2016; McGill, Enquist, Weiher, & Westoby, 2006). Specifically, plant size/growth is often positively correlated with reproductive output and competitiveness (Ackerly & Monson, 2003; Younginger, Sirová, Cruzan, & Ballhorn, 2017), and variation in leaf morphology can summarize differences in plant ecological strategies along a leaf economic spectrum (Wright et al., 2004). Whereas Stahl et al. (2014) used multiple species ranges that varied in occurrence probability (i.e., some relatively dominant and others relatively rare), we use a single‐species range with sixteen distinct watersheds (each encompassing an elevation gradient) along the overall species‐level occurrence probability distribution. We calculated mean trait and climate values at upper, middle, and lower sites along the elevational transects (Figure 1). We then constructed double quantile regressions for each trait‐climate combination using the “rq” function in the quantreg package in R (Koenker, 2016). We also standardized individual trait and climate data using *z*‐scores to compare different trait‐climate responses patterns. Slopes from trait‐climate correlations that differ from zero at the species' central environments (50th and 55th quantile) show a relationship between functional traits and the median climatic niche. More importantly, we interpret slopes that differ from zero at upper limits (90th and 95th) or lower limits (5th and 10th) as support for our first hypothesis by showing how functional trait variation predicts climate boundaries.

The space between the outer quantiles reflects the climate niche that *P. angustifolia* can occupy across the range of trait measurements, with climate habitats beyond these limits acting as “no‐go areas” for trees with certain trait values (Stahl et al., 2014). Therefore, the response pattern shapes (the area between the outer quantile) are informative for understanding how functional trait variation corresponds to the species' climate range:

- *Aligned* (rhombus)—slopes from upper and lower trait‐climate relationships are significant in the same direction, creating a rhombus shape where a change in trait value does not correspond to a change in the climate range of the species.
- *One‐sided* (wedge)—the slope of either the upper or lower quantile is significant, creating a wedge shape that indicates a constraint on trait values at one climate extreme.
- *Reverse* (acute triangle)—both outer quantiles have significant slopes in opposing directions, creating an acute triangle shape that reflects a double‐sided constraint on traits from both ends of a climate gradient.

One‐sided and reverse patterns (II and III) show how the possible climate range of *P. angustifolia* can change as trait values increase or decrease.

To test the second hypothesis (that genetic variation from traits in the common garden experiment will predict climate range limits), we used the same double quantile approach described above using common garden traits (annual growth diameter, leaf area, leaf mass, SLA). Similar response patterns between the trait‐environment relationships in the field and common garden would suggest that genetic clines influence functional trait variation at climate range limits (Connor & Hartl, 2004). In contrast, different response patterns between field and common garden trait‐climate relationships would be consistent with genetic × environment interactions and phenotypic plasticity at climate range limits. We also include multivariate analyses to further test how double quantile regression may be useful to determine trait‐environment relationships considering a combination of climate pressures (see Appendix S1: *Multivariate analysis of trait‐environment relationships*; Figure S2).

### 2.4 Mapping constraints on functional trait variation

We modeled the associations between limits of *P. angustifolia* trait values and climate gradients by applying the quantile regression equations to gridded climate data. We used only ENVIREM climate layers where consistent response patterns were found for both field and common garden trait‐climate relationships. We selected the lowest trait values per grid cell to identify the strongest climatic constraints (sensu Stahl et al., 2014), and then projected the resulting trait variation across the climate range occupied by *P. angustifolia* in the western US. The resulting maps show geographic variation in maximum attainable trait values (based on our sites and samples) and provide spatially explicit patterns of constraints on genetic variation in functional traits across climate gradients. All analyses were performed in R version 3.4.1 (R Core Team, 2016).

## 3 RESULTS

### 3.1 Trait‐climate range response patterns

**Figure 2:**
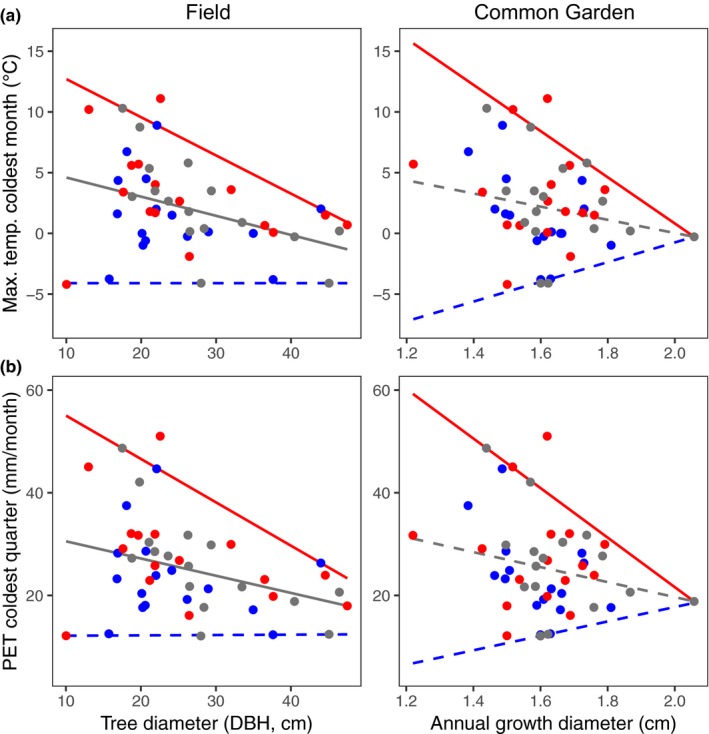
Trait‐climate response patterns show that plant size and growth may be adapted to the upper limits of maximum temperature and PET during winter months. (a) The upper limit (95th quantile) of maximum temperature during the coldest quarter was positively correlated with *P. angustifolia* DBH in the field and the diameter of new shoot growth of cuttings in the common garden. (b) We found a similar pattern when using these traits to predict the amount of potential evapotranspiration (PET) during the coldest quarter. Red = 95th quantile, gray = 50th quantile, blue = 5th quantile; solid lines represent significant slopes, dashed lines represent nonsignificant slopes

**Figure 3:**
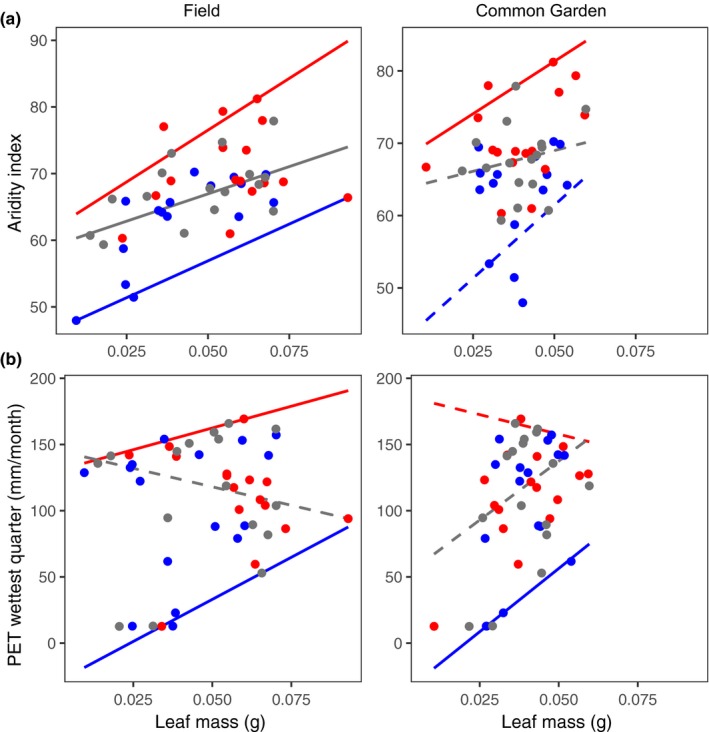
Leaf mass is adapted to the upper limits of aridity and lower limits of PET during the wettest quarter. (a) Leaf mass in the field positively correlated with the upper (95th), median (50th), and lower (5th) limits of aridity, resulting in an aligned response pattern. Leaf mass in the common garden positively correlated to the upper limits (95th quantile) of aridity. (b) Leaf mass showed patterns of adaptation to the lower limits of PET during the wettest quarter. Red = 95th quantile, gray = 50th quantile, blue = 5th quantile; solid lines represent significant slopes, dashed lines represent nonsignificant slopes

In support of Hypothesis 1, *P. angustifolia* functional trait variation predicts the species' climate range using double quantile regressions. The most common type of response pattern differed among field traits (Tables S2 and S3). Nearly two‐thirds (9/15) of the response patterns from DBH‐climate quantile regressions were one‐sided (i.e., wedge shaped). This indicates that the climate range of *P. angustifolia* changes with a given DBH value, and that tree diameter is generally constrained at either an upper or lower climate limit (Figure S3). For instance, larger DBH predicted declines in the upper limits of the maximum temperature of the coldest month (Figure 2a) and PET of the coldest quarter (Figure 2b). Aligned response patterns were most common for leaf area (7/15; Figure S4) and leaf mass (11/15; Figure S5) (Tables S2 and S3), indicating that these traits predict a shift in mean climate niche for *P*. *angustifolia* genotypes in the field. As an example, leaf mass positively predicted the upper and lower limits of aridity (Figure 3a) and PET during the wettest quarter (Figure 3b). There were fewer obvious SLA‐climate response patterns than for the other three traits, with only four one‐sided patterns, two reverse patterns, and one aligned pattern (Tables S2 and S3, Figure S6).

In support of our second hypothesis, common garden traits of *P. angustifolia* clonal cuttings (with variation primarily controlled by plant genetic effects) predict the species' climate range and show that genetic variation in functional traits is constrained at the species' climate limits. Annual growth diameter showed three obvious one‐sided response patterns that match the same patterns from DBH‐climate regressions (Tables S2 and S3): continentality (5th quantile; Figure S3), maximum temperature of the coldest month (95th quantile; Figure 2a), and PET of the coldest quarter (95th quantile; Figure 2b). Although most leaf area and leaf mass response patterns in the field were aligned, they primarily showed one‐sided response patterns in the common garden (Tables S2 and S3, Figures S4 and S5). For example, genetic variation in leaf mass predicted the upper limits of aridity (Figure 3a), and lower limits of PET of the wettest quarter (Figure 3b). These are consistent trends with leaf mass variation from the field and show a constraint on genetic variation at these climatic limits. In contrast, genetic variation in leaf mass was unrelated to the lower limits of aridity and upper limits of PET of the wettest quarter, consistent with phenotypic plasticity in leaf mass values at these climate extremes. Genetic variation in SLA‐climate relationships produced only one‐sided response patterns (Table S2), but the type (upper or lower) and direction of the quantile regressions differed from field SLA‐climate relationships (Table S3, Figure S6).

### 3.2 Mapping constraints on functional trait variation

**Figure 4:**
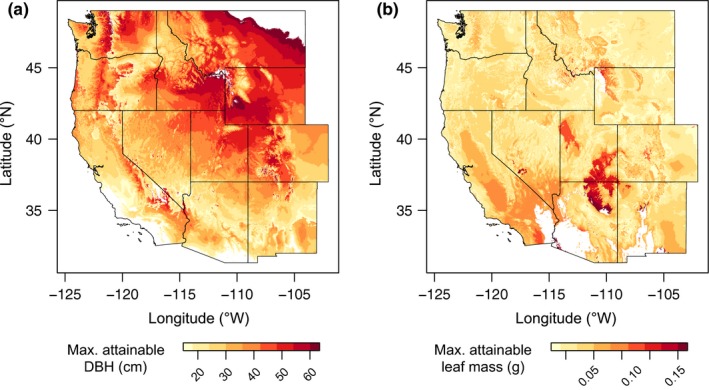
Patterns of genetic clines in functional traits at climate range limits show geographic variation in maximum attainable trait values. Parameter estimates from quantile regression equations were applied to gridded climate data when both field and common garden trait‐climate regressions were significant in the same direction (Tables S4 and S5). We selected the minimum trait values per grid cell to visualize the strongest climate constraints on (a) tree diameter (DBH) and (b) leaf mass variation. The darkest regions show areas where traits are climatically unconstrained. Moving from darker to lighter areas shows how climate range limits restricts the maximum attainable trait values across the landscape (i.e., “no‐go areas” for *P. angustifolia* genotypes with certain trait values). Climate data used for DBH: continentality, maximum temperature of the coldest quarter, and PET of the coldest quarter. Climate data used for leaf mass: aridity index, maximum temperature of the coldest quarter, minimum temperature of the warmest quarter, and PET of the wettest quarter. White areas are regions outside the phenotypic or climate range of *P. angustifolia* and are not included in the prediction

We modeled functional trait responses to climate by applying double quantile regressions to gridded climate data (Tables S4 and S5). By using only response patterns that were consistent between field and common garden trait‐climate relationships, the color gradient in Figure 4 represents the quantitative genetic component of *P. angustifolia* trait responses to climate as maximum trait values decrease along climatic gradients. The darkest areas indicate where trait variation is genetically unconstrained (i.e., all observed values of a given trait are possible in this climate). We find that tree diameter is predicted to be unconstrained at higher latitudes with areas in Montana and Wyoming having climates suitable for the widest range of *P. angustifolia* DBH values (<20 cm to >60 cm). From here, “no‐go” areas for larger trees extend southward until ~20 cm is expected to be the maximum attainable DBH for trees in central Arizona along the Mogollon Rim (Figure 4a). We also find that arid regions in northern Arizona and southern Utah should allow for the greatest range of leaf mass values from <0.05 to >0.15 g (i.e., genetic clines in leaf mass are largely unconstrained by climate). However, most other areas within or near the species' range exert some climatic pressure that define “no‐go areas” for genotypes with leaf masses greater than ~0.05 g (Figure 4b). These examples demonstrate that the double quantile regression approach can be used to make spatially explicit predictions of how genetic clines in functional traits respond to climate.

## 4 DISCUSSION

Accurately forecasting plant species distributions and range limits are a central challenge for understanding climate change effects on the landscape. However, most ENM fails to include information on the ecophysiological mechanisms that make habitats suitable for plants in the first place. Here, we extend a recently proposed quantile regression approach using within‐species functional trait variation across *P. angustifolia* elevation gradients to predict a single species' climate niche. We tested and found evidence that extreme values of tree size and leaf morphology correspond with the species' climate range limits. Moreover, using a common garden experiment to isolate the genetic effects on trait variation, we found evidence of genetic clines in leaf mass at climatic limits, but also many patterns consistent with phenotypic plasticity at the tree species' environmental boundaries. Finally, we applied the quantile regression models in reverse to climate data to geographically predict how functional trait variation is constrained by the species' climate niche limits across *P. angustifolia* habitat in the western United States. Collectively, these findings point toward the ecological and evolutionary drivers of climate range limits and demonstrate how this approach can complement current ENM to improve their accuracy by including intraspecific trait‐climate responses.

### 4.1 Trait‐climate range response patterns reveal genetic and plastic responses at range limits

Variation in plant functional traits predicts the environmental range and climate limits of a dominant tree species (Hypothesis 1), which fit well‐studied mechanisms behind plant physiological and morphological responses across temperature and moisture gradients. Variation in tree DBH largely predicted the species' warm, dry climate range limits. Our double quantile regression analysis showed significant DBH‐climate trends at the 95th quantiles of annual PET, growing degree days above 0°C, maximum temperature of the coldest month, month by temperature above 10°C, PET of the warmest and coldest quarter, and thermicity. The response patterns were all one‐sided (wedge shaped), indicating that DBH variation is constrained by these upper climate extremes and smaller trees can occur in a wider range of climate habitats. Tree productivity is strongly regulated by water deficit levels across the globe (Babst et al., 2019; Zhang, Niinemets, Sheffield, & Lichstein, 2018), and in *Populus* specifically (Heilman, Hinckley, Roberts, & Ceulemans, 1996). Consistent with this work, our results show that the drier limits of temperature and moisture‐related climate gradients are likely predominant factors imposing physiological limits on *P. angustifolia* tree size.

Leaf mass and leaf area are two key traits related to the investment strategies of plants faced with building robust leaves versus maximizing photosynthetic area (Wright et al., 2004). For instance, plants with thick leaves are often found in arid conditions to maintain photosynthesis while reducing transpirational water loss (Wright et al., 2004). Our results show that leaf mass variation (likely covarying with leaf thickness; Marron et al., 2005; Niinemets, 2001) strongly predicts temperature and moisture climate limits, including aridity, annual PET, PET of the driest and warmest quarters, and thermicity. Moreover, SLA variation showed similar patterns, demonstrating that this integrated trait seems to capture the expected leaf strategy tradeoffs occurring at arid climatic niche limits. Greater leaf mass for *P. angustifolia* in arid sites might also result from the combination of tolerating water scarcity and herbivory: thicker leaves should prevent excessive transpirational loss (Heilman et al., 1996) and provide more robust defense against greater herbivore pressures in warmer sites (Onoda et al., 2011; Pennings & Silliman, 2005; Whitham et al., 1996). Further work is needed to test how the physiological processes controlling foliar anatomy might interact with nonclimatic pressures to affect leaf growth strategies across environmental gradients.

Climate can impose selective pressures on heritable traits (Ware et al., 2019) such that genetically based trait‐climate response patterns might hint at the evolutionary dynamics occurring at species' niche limits. We found that common garden trait‐climate relationships showed some similar response patterns as field traits (Hypothesis 2), indicating the presence of genetic clines that constrain trait variation at climate niche limits. For example, variation in tree DBH (field) and annual growth (common garden) predicts the upper limits of the maximum temperature of the coldest month and PET of the coldest quarter. Interestingly, these results contrast with recent work showing that temperature positively correlates with aboveground biomass and spring phenology (Ware et al., 2019), suggesting a tradeoff between the stress of growing in warm, dry climates versus the benefit of longer growing seasons. Leaf mass showed the greatest number of significant quantile regressions that were consistent between field and common garden comparisons. This includes genetically based trait responses to aridity (95th quantile), maximum temperature of the coldest month (5th quantile), month by temperature above 10°C (5th quantile), minimum temperature of the warmest month (5th quantile), PET of the coldest quarter (5th quantile), and PET of the wettest quarter (5th quantile). Together, these results are consistent with trait adaptations where environmental selection acts to constrain niche evolution at range boundaries (Angert, 2009; Grady et al., 2011).

We also found many cases that are more consistent with plastic trait responses to climate (i.e., trait variation at range limits explained by environment, not genetic effects). The number of inconsistent patterns between field and common garden trait‐climate relationships suggests that some level of phenotypic flexibility in plant growth and leaf morphology helps genotypes persist at contemporary climatic limits (Valladares et al., 2014). It is also possible that the context of field versus common garden measurements of functional traits affected these comparisons (e.g., mature trees vs. rooted cuttings, tree size vs. annual growth), or that functional plasticity itself could be an adaptive response to variable climate pressures (Aitken et al., 2008; Gunderson et al., 2010). Nonetheless, the genetically based trait‐climate relationships we identified in this study may have important evolutionary consequences for understanding *P. angustifolia* responses to environmental change.

### 4.2 Mapping constraints on functional trait variation will improve species range forecasts

Our study demonstrates a new integrative approach (double quantile regression with a single species, elevation gradients, and a common garden experiment) that can greatly complement and improve upon existing ENM. We modeled how genetic clines in *P. angustifolia* growth traits are constrained by applying quantile regressions that were significant for DBH (field) and annual growth diameter (common garden) to gridded climate data. When projected across the western US, we find that variation in growth decreases from north‐to‐south (i.e., smaller trait‐space between upper and lower quantile limits), and the maximum attainable DBH values decline from Montana to Arizona. In other words, the climate gradients covering these portions of the species range gradually impose “no‐go areas” for genotypes with growth traits above a certain DBH threshold, which decreases the potential range of trait values by limiting the maximum attainable DBH. This pattern suggests that southern populations may have insufficient genetic variation in growth traits for adaptive evolutionary responses to climate change (Becklin et al., 2016), which is consistent with recent work showing that warm, southern populations of *P. angustifolia* have reduced adaptive potential and evolvability (Ware et al., 2019). Riparian habitats in the western US are predicted to warm by 2–6°C in the next 50 years (Capon et al., 2013; Van Nuland, Bailey, & Schweitzer, 2017), and populations that are adapted to current climate limits may become maladapted if temperature rises and population‐mean trait values no longer confer optimal fitness.

We also project that leaf mass variation is largely unconstrained in some of the most arid regions of the species' distribution (Northern Arizona and Southern Utah), but that most other areas in the western US have at least one major climate pressure that constrains the lower limits of leaf mass variation. In summary, our approach shows the advantages in using double quantile regression for creating geographic predictions of genetically based functional trait variation. We demonstrate this for the first time using a single widespread *Populus* tree species, but it should be noted that other studies exist with similar sampling designs for which this method could be applied with *Clarkia* (Jonas & Geber, 1999), *Mimulus* (Kooyers, Greenlee, Colicchio, Oh, & Blackman, 2015), *Rhododendron* (Pfennigwerth, Bailey, & Schweitzer, 2017), and *Solidago* (Moran, Reid, & Levine, 2017), among others. It should be noted that estimates of maximum attainable trait values are based on sites and measurements specific to this study. Even though our sampling approach was intended to capture as much climatic and phenotypic variation as possible, larger sampling efforts or experimental considerations of novel trait‐climate combinations might alter the total range of trait variation. Regardless, these projections are based on the ecophysiological mechanisms that help shape species' climate range limits and, therefore, can be used to directly improve the accuracy of ENM that forecast future species distributions by incorporating information about how genetic variation in functional traits respond to climate (Ikeda et al., 2014, 2017).

### 4.3 Remaining opportunities

Our study demonstrates how a widespread tree species' climate range is predicted by intraspecific trait variation. Because phenotypes are the level upon which plants physically respond and interact with their climatic (Wright et al., 2004), biotic (McGill et al., 2006; Tilman, 1982), and edaphic environments (Chapin, 2003; van der Putten et al., 2013), we highlight three opportunities and challenges for expanding these methods to improve our mechanistic understanding of how plant traits influence species distributions along environmental gradients. First, tree populations may not be in equilibrium with their current climate (Svenning & Skov, 2004), and some amount of functional trait variation could reflect the evolutionary history of past climate stress. Using the approach described here, it seems possible to determine how trait‐climate responses have changed over time using functional trait measurements from georeferenced herbarium specimens paired with historical climate data. Second, geographic predictions of trait variation could be directly incorporated with ENM to identify potential environmental filtering mechanisms (Stahl et al., 2014; Valladares et al., 2014), or in joint species distribution models characterizing biotic interactions that mediate species cooccurrence and community assembly patterns (Pollock, Morris, & Vesk, 2012; Pollock et al., 2014). However, caution should be exercised with predictions here as trait variation is affected by biotic and climatic interactions that may also shift with changing conditions. Third, plant traits and geographic distributions are, in part, shaped by soil physical, chemical, and biological characteristics. For example, SLA correlates with soil fertility at the global scale (Ordoñez et al., 2009), and soil factors can be important predictors of habitat suitability (Beauregard & de Blois, 2014; Coudun, Gegout, Piedallu, & Rameau, 2006). While it is beyond the scope of the present study, incorporating soil gradients into the double quantile approach is particularly important since plant traits both respond to and alter soil environments (Chapin, 2003; van der Putten et al., 2013), creating feedback effects that might boost or reduce plant performance at environmental limits (Van Nuland et al., 2017; Ware et al., 2019).

## 5 CONCLUSIONS

Plant species across the globe are encountering multifaceted changes to their habitats, requiring a variety of relevant tools and information to preserve and manage these environments. Our study shows that patterns from the double quantile regression approach could be used in conjunction with traditional ENM to improve their accuracy for predicting the location and extent of future climate suitability. Plant trait‐climate relationships that occur at environmental extremes (such as the ones identified for growth traits and leaf economic strategies in this study) are indicative of the physiological and evolutionary mechanisms that influence species range limits. Pairing this approach with ongoing advancements in ecological forecasting that includes an explicit consideration of genetic variation will significantly improve our ability to predict the adaptive capacity and local extirpation risk of marginal populations under future climate scenarios.

## CONFLICT OF INTEREST

None declared.

## AUTHOR CONTRIBUTIONS

MVN and JBV conceived the original ideas, analyzed the data, and wrote the first draft of the manuscript; IMW, LOM, SLJB, KKB, JAS, and JKB all contributed to the discussion of ideas and provided substantial edits to the manuscript.

## Data Availability

All data and code related to this manuscript have been archived and are freely available on Zenodo (https://doi.org/10.5281/zenodo.3571431).
