# Supplementary material for: Intraspecific trait variation across elevation predicts a widespread tree species' climate niche and range limits
Source: Ecol Evol. 2020 Apr 17;10(9):3856–67. doi: 10.1002/ece3.5969 (PMC7244802; doi:10.1002/ece3.5969)
Supplement: Supplementary file 1 [file ECE3-10-3856-s001.docx]

SUPPLEMENTAL INFORMATION

**Intraspecific trait variation across elevation predicts a widespread tree species’ climate niche and range limits**

Michael E. Van Nuland^1*^, John B. Vincent^2^, Ian M. Ware^3^, Liam O. Mueller^4^, Shannon L. J. Bayliss^4^, Kendall K. Beals^4^, Jennifer A. Schweitzer^4^, and Joseph K. Bailey^4^

^1^ Department of Biology, Stanford University, Stanford, CA 94305, USA

^2^ University of Washington, School of Environmental and Forest Sciences, Seattle, WA 98195

^3^ Institute of Pacific Islands Forestry, USDA Forest Service, Pacific Southwest Research Station, Hilo, HI, 96720

^4^ University of Tennessee, Department of Ecology and Evolutionary Biology, Knoxville, TN 37996

* Corresponding author (mvannula@stanford.edu)

Contents:

**Appendix S1:** *Multivariate analysis of trait-environment relationships*

**Table S1**. Correlation results of climate variation across elevation transects.

**Table S2**. Total number of each response pattern type from the trait-climate quantile regressions.

**Table S3**. Standardized slopes of quantile regression lines for trait-climate range relationships.

**Table S4**. Intercept and slope parameter estimates of significant quantile regressions for field trait-climate range models.

**Table S5**. Intercept and slope parameter estimates of significant quantile regressions for common garden trait-climate range models.

**Figure S1**. Trait variation across elevation gradients.

**Figure S2**. Trait-climate range relationships using principle component analysis.

**Figure S3**. Plant size and growth trait-climate range quantile regressions.

**Figure S4**. Leaf area-climate range quantile regressions.

**Figure S5**. Leaf mass-climate range quantile regressions.

**Figure S6**. Specific leaf area-climate range quantile regressions.

*Multivariate analysis of trait-environment relationships*

Plants experience a range of climate gradients that act together to shape functional trait variation. As a result, we tested if field and common garden traits would predict such a combined environmental gradient using multivariate analysis. We first combined the ENVIREM variables used in quantile regression analysis with the ‘rda’ function in the vegan package. The first PCA axis explained 61.4% of the total variance in climate among all sampling locations (Figure S2A). Next, we identified PCA axis 1 values across elevation in the same manner as ENVIREM climate variables and functional traits by calculating the average value at the highest, middle, and lowest position along the elevation transect in each sampled watershed. Using the double quantile approach (described in main text), we tested whether field and common garden traits predicted variation in these average values of PCA Axis 1. Identical to the individual trait-environment relationships for single climate variables, slopes that differed from zero at the 5^th^ or 95^th^ quantiles would indicate that *Populus angustifolia* functional trait variation predicts the range of combined environmental gradients. Moreover, consistent response patterns across both field and common garden comparisons would suggest that traits may be adapted to such combined environmental pressures at range limits.

We find that thermicity, annual PET, growing days above 0C, month by temperature 10C, growing days above 5C, PET of the warmest quarter, minimum temperature of the warmest month, maximum temperature of the coldest month, and PET of the coldest quarter are the most important factors contributing to the variance explained by PCA axis 1 (Figure S2A). Using double quantile regression, we find that DBH and leaf trait variation from field measurements do predict the species’ combined climate niche and range limits. DBH-climate patterns showed a one-sided constraint at upper limits, and leaf area and mass both showed aligned patterns (Figure S2B). In contrast, there are no consistent patterns between field and common garden trait-climate relationships using ENVIREM PCA axis 1, apart from median niche (50^th^ quantile) correlations in leaf area and mass (Figure S2C). Together, these results illustrate that the expression *P. angustifolia* growth and foliar traits in the field are related to the combined climatic limits of the species distribution, though it is less obvious how patterns of plasticity or genetic differentiation might be structured using the combined climate compared to relationships of single trait-climate comparisons.

**Table S1**. Correlation results of climate variation across elevation transects.

Elevation

ENVIREM variable r^2^ p-value Direction

Climate moisture index 0.11 <0.001 Positive

Aridity 0.18 <0.001 Negative

Annual PET 0.13 <0.001 Negative

Continentality 0.07 <0.001 Negative

Grow deg. days 0°C 0.32 <0.001 Negative

Grow deg, days 5°C 0.36 <0.001 Negative

Max. temp. coldest 0.03 <0.001 Negative

Month by temp. 10°C 0.36 <0.001 Negative

Min. temp. warmest 0.39 <0.001 Negative

PET driest quarter 0.05 <0.001 Negative

PET coldest quarter 0.02 0.004 Negative

PET warmest quarter 0.39 <0.001 Negative

PET wettest quarter 0 0.74 N/A

PET seasonality 0.59 <0.001 Negative

Thermicity 0.38 <0.001 Negative

**Table S2**. Total number of each response pattern type from the trait-climate quantile regressions.

Plant traits Location Aligned One-sided Reverse

Diameter Field 0 9 0

Growth Common garden 0 3 0

Leaf area Field 7 2 0

Leaf area Common garden 0 3 0

Leaf mass Field 11 1 0

Leaf mass Common garden 1 5 0

SLA Field 1 4 2

SLA Common garden 0 5 0

Totals refer to obvious response patterns (as in Stahl et al. 2014) where upper quantiles (90th and 95^th^) and/or lower quantiles (5^th^ and 10^th^) have slopes that are significantly different from zero. Aligned response patterns indicate no change in climate range per trait value. One-sided response patterns reflect a constraint at either the upper or lower climate limit. Reverse response patterns indicate constraints at both upper and lower climate limits. Growth = annual growth diameter, SLA = specific leaf area.

**Table S3**. Standardized slopes of quantile regression lines for trait-climate range relationships.

*Field traits* *Common garden traits*

ENVIREM DBH Leaf Area Leaf Mass SLA Growth Leaf Area Leaf Mass SLA

Clim. moist. index O-S O-S O-S Aligned n/a n/a n/a n/a

95th quantile -0.37 -0.39 -0.41 0.48 -0.13 -0.22 -0.14 -0.33

90th quantile -0.10 -0.49^**^ -0.62^**^ 0.57^*^ -0.16 -0.29 -0.51 0.06

55th quantile 0.04 -0.54^*^ -0.63^**^ 0.27 0.07 -0.55^**^ -0.50^***^ 0.36^*^

50th quantile 0.03 -0.60^*^ -0.60^**^ 0.31 0.03 -0.38^*^ -0.44^***^ 0.40^*^

10th quantile 0.42^*^ -0.22 -0.35 0.35^*^ -0.11 -0.09 -0.15 0.10

5th quantile 0.27 -0.07 -0.41 0.37^**^ 0.00 -0.19 -0.19 -0.02

Aridity n/a **O-S Aligned O-S** n/a O-S  **O-S** n/a

95th quantile -0.55 0.25 0.87^**^ -0.55^*^ 0.05 0.44^*^ 0.44^*^ -0.11

90th quantile -0.41 0.79 0.71^*^ -0.50^*^ 0.23 0.33 0.42^*^ -0.21

55th quantile 0.08 0.20 0.41^*^ -0.40 0.11 0.17 0.22 -0.08

50th quantile 0.10 0.18 0.46^**^ -0.48^*^ 0.08 0.17 0.18 -0.16

10th quantile 0.08 0.40^*^ 0.68^**^ -0.40 0.52 0.34 0.52 0.35

5th quantile 0.49 0.63^**^ 0.62^***^ -0.41 0.58 0.56 0.62 0.90

Annual PET **O-S Aligned Aligned O-S** O-S O-S O-S  **O-S**

95^th^ quantile -0.65^**^ 1.05^**^ 1.11^***^ -0.88^*^ -0.39 -0.28 -0.30 0.15

90^th^ quantile -0.49^**^ 0.63^*^ 0.82^***^ -0.86^*^ -0.46^*^ 0.31 0.27 0.08

55^th^ quantile -0.32 0.48^*^ 0.61^**^ -0.26 -0.23 0.38^*^ 0.35^*^ -0.04

50^th^ quantile -0.29 0.42^*^ 0.48^*^ -0.19 -0.13 0.36^*^ 0.37^*^ 0.16

10^th^ quantile 0.02 0.37^***^ 0.45^***^ 0.10 0.06 0.44^*^ 0.44^*^ -0.35^*^

5^th^ quantile -0.04 0.51^***^ 0.45^***^ 0.01 0.07 0.14 0.17 -0.43^**^

Continentality **O-S** n/a n/a **O-S O-S** n/a n/a n/a

95^th^ quantile 0.05 0.11 0.13 -0.44^*^ 0.22 0.06 0.06 -0.06

90^th^ quantile 0.01 0.18 0.19 -0.35^*^ 0.07 -0.02 -0.02 0.04

55^th^ quantile 0.30 0.16 0.36^*^ -0.18 0.29 -0.03 0.07 -0.24

50^th^ quantile 0.34 0.19 0.32 -0.19 0.24 -0.07 0.00 -0.22

10^th^ quantile 0.83^***^  -0.20 -0.21 0.24 0.59^***^ -0.45 -0.19 -0.18

5^th^ quantile 0.66^***^ -0.21 -0.22 0.32 0.65^**^ -0.20 -0.19 0.10

(continued on next page)

*Field traits* *Common garden traits*

ENVIREM DBH Leaf Area Leaf Mass SLA Growth Leaf Area Leaf Mass SLA

Grow days 0°C **O-S Aligned Aligned** n/a O-S O-S n/a **O-S**

95^th^ quantile -0.73^***^ 0.94^**^ 1.20^**^ 0.18 -0.63^*^ 0.97^*^ 0.29 0.53^*^

90^th^ quantile -0.71^***^ 0.97^***^ 1.02^**^ -0.22 -0.38 0.67 -0.05 0.47^*^

55^th^ quantile 0.03 0.52^***^ 0.56^***^ -0.40^**^ -0.05 0.33^*^ 0.33^*^ 0.04

50^th^ quantile 0.04 0.47^***^ 0.54^***^ -0.34^**^ -0.06 0.43^*^ 0.34^*^ 0.03

10^th^ quantile 0.06 0.24^*^ 0.31^**^ -0.10 0.10 0.13 0.11 -0.08

5^th^ quantile -0.07 0.27^**^ 0.33^***^ -0.07 -0.02 0.13 0.15 -0.12

Grow days 5°C O-S **Aligned** **Aligned** n/a n/a n/a n/a O-S

95^th^ quantile -0.72^**^ 1.34^***^ 1.22^**^ -0.12 -0.77 0.55 -0.19 0.68^*^

90^th^ quantile -0.49 0.95^**^ 0.95^**^ -0.42 -0.20 0.49 0.12 0.42

55^th^ quantile 0.02 0.52^***^ 0.54^***^ -0.42^***^ -0.05 0.39^**^ 0.38^*^ -0.03

50^th^ quantile 0.05 0.48^***^ 0.59^***^ -0.37^***^ -0.12 0.41^*^ 0.40^*^ -0.01

10^th^ quantile 0.05 0.29^***^ 0.41^***^ -0.05 0.10 0.22 0.19 -0.17

5^th^ quantile 0.00 0.37^***^ 0.41^***^ -0.03 -0.02 0.08 0.10 -0.01

Max. temp. coldest **O-S Aligned Aligned Reverse O-S** O-S **O-S O-S**

95^th^ quantile -0.78^***^ 0.69^*^ 0.86^***^ -0.68^*^ -0.70^**^ -0.03 -0.33 0.15

90^th^ quantile -0.72^***^ 0.63^**^ 0.64^**^ -0.73^*^ -0.63^**^ 0.58 0.36 0.42

55^th^ quantile -0.38 0.35 0.34 0.02 -0.22^*^ 0.26 0.11 -0.06

50^th^ quantile -0.39 0.29 0.31 0.03 -0.20 0.34^*^ 0.17 0.01

10^th^ quantile -0.29 0.40^**^ 0.43^**^ 0.34^**^ 0.26 0.41^*^ 0.50** -0.50^**^

5^th^ quantile 0.00 0.25^*^ 0.50^***^ 0.35^**^ 0.30 0.25 0.37** -0.47^**^

Month by temp. 10°C **O-S Aligned Aligned** n/a n/a O-S **O-S O-S**

95^th^ quantile -0.48^**^ 0.76^***^ 0.81^**^ 0.00 -0.35 0.41 0.18 0.28^*^

90^th^ quantile -0.31^*^ 0.83^***^ 0.77^**^ -0.07 0.00 0.58^*^ 0.06 0.32^*^

55^th^ quantile 0.00 0.47^*^ 0.48^**^ -0.36^*^ 0.00 0.47^*^ 0.38 0.00

50^th^ quantile 0.00 0.56^***^ 0.59^***^ -0.36^**^ 0.00 0.46^*^ 0.46^*^ 0.00

10^th^ quantile 0.00 0.37^*^ 0.61^**^ -0.14 0.23 0.43 0.40^*^ -0.41

5^th^ quantile -0.24 0.54^*^ 0.77^***^ -0.28 0.09 0.49 0.56^*^ -0.49

(continued on next page)

*Field traits* *Common garden traits*

ENVIREM DBH Leaf Area Leaf Mass SLA Growth Leaf Area Leaf Mass SLA

Min. temp. warmest n/a **Aligned** **Aligned** n/a n/a **O-S** **Aligned** n/a

95^th^ quantile -0.32 0.97^***^ 0.60^*^ -0.11 0.21 0.35^*^ 0.43^*^ 0.12

90^th^ quantile -0.26 0.77^***^ 0.98^***^ 0.17 0.11 0.38^*^ 0.33 0.18

55^th^ quantile -0.02 0.69^***^ 0.78^***^ -0.44^***^ -0.05 0.50^*^ 0.34 0.04

50^th^ quantile -0.01 0.74^***^ 0.82^***^ -0.38^**^ -0.01 0.46^*^ 0.42^*^ -0.02

10^th^ quantile 0.09 0.48^**^ 0.70^***^ -0.01 0.34 0.36 0.35^*^ -0.32

5^th^ quantile -0.08 0.59^***^ 0.71^***^ 0.07 0.45 0.34 0.38^*^ -0.18

PET driest quarter n/a **O-S**  **O-S**  **O-S**  n/a n/a n/a O-S

95^th^ quantile 0.05 0.25^**^ 0.32^***^ -0.39^**^ 0.07 0.14 0.17 0.20^*^

90^th^ quantile 0.07 0.22^***^ 0.28^***^ -0.34^**^ 0.11 0.15 0.17 0.12

55^th^ quantile 0.04 0.24^**^ 0.23^*^ -0.12 0.11 0.12 0.02 0.14

50^th^ quantile 0.12 0.23^*^ 0.25^*^ -0.12 0.18 0.20 0.12 0.18

10^th^ quantile -0.06 0.06 -0.01 0.14 0.00 -0.03 -0.03 0.02

5^th^ quantile -0.10 0.06 -0.12 0.09 0.00 -0.11 -0.11 0.06

PET cold. quarter **O-S** Aligned **Aligned Reverse O-S O-S O-S** O-S

95^th^ quantile -0.84^***^ 0.77^*^ 1.07^***^ -0.84^*^ -0.71^*^ -0.44 -0.38 0.02

90^th^ quantile -0.73^***^ 0.64^***^ 0.66^*^ -0.79^*^ -0.67^**^ 0.79 0.41 0.35

55^th^ quantile -0.37^**^ 0.29 0.29 -0.09 -0.23 0.30 0.14 -0.09

50^th^ quantile -0.33^*^ 0.20 0.29 -0.04 -0.22 0.37^*^ 0.25 -0.09

10^th^ quantile -0.20 0.31^**^ 0.34^***^ 0.28^**^ 0.18 0.29^*^ 0.28^*^ -0.26

5^th^ quantile 0.01 0.17 0.24^**^ 0.30^**^ 0.21 0.19^*^ 0.27^**^ -0.34^*^

PET warm. quarter **O-S** O-S  **Aligned** Aligned n/a n/a n/a n/a

95^th^ quantile -0.34^*^ 0.75 0.83^***^ -0.32 0.00 0.18 0.21 -0.04

90^th^ quantile -0.33^*^ 0.66 0.95^***^ -0.63^**^ -0.02 0.18 0.05 0.05

55^th^ quantile -0.17 0.63^***^ 0.78^***^ -0.54^**^ -0.08 0.41^*^ 0.32 0.18

50^th^ quantile -0.17 0.60^***^ 0.75^***^ -0.47^**^ -0.04 0.56^**^ 0.32 0.20

10^th^ quantile 0.24 0.55^***^ 0.63^***^ -0.41^**^ 0.19 0.10 0.12 0.05

5^th^ quantile 0.11 0.41 0.72^***^ -0.32^*^ 0.24 0.12 0.18 0.17

(continued on next page)

*Field traits* *Common garden traits*

ENVIREM DBH Leaf Area Leaf Mass SLA Growth Leaf Area Leaf Mass SLA

PET wet. quarter n/a Aligned **Aligned** Reverse O-S **O-S O-S**  **O-S**

95^th^ quantile -0.14 0.33^*^ 0.27^***^ -0.19^*^ -0.14 -0.07 -0.13 0.02

90^th^ quantile -0.16 0.13 0.16^*^ -0.19 -0.17^*^ 0.11 0.04 0.06

55^th^ quantile -0.07 -0.04 -0.18 0.25^*^ -0.32^*^ 0.07 0.38 -0.33^*^

50^th^ quantile -0.04 -0.07 -0.24 0.25^*^ -0.27^*^ 0.26 0.39 -0.40^*^

10^th^ quantile -0.11 0.39^*^ 0.53^**^ 0.82^*^ 0.39 0.46^*^ 0.72^***^ -0.74^**^

5^th^ quantile 0.00 0.37^**^ 0.51^***^ 0.21 0.02 0.36^*^ 0.42^*^ -0.79^**^

PET seasonality **O-S** Aligned Aligned **Aligned** n/a n/a O-S n/a

95^th^ quantile -0.17 0.79^*^ 0.93^***^ -0.68^*^ 0.01 0.29 0.32^*^ -0.02

90^th^ quantile -0.03 0.76^***^ 0.83^***^ -0.67^***^ -0.03 0.24 0.23 -0.01

55^th^ quantile 0.12 0.47^**^ 0.72^***^ -0.60^**^ 0.20 0.04 0.17 -0.02

50^th^ quantile 0.07 0.43^**^ 0.65^***^ -0.54^**^ 0.19 0.18 0.23 0.04

10^th^ quantile 0.51^*^ 0.39^*^ 0.57^*^ -0.72^**^ 0.31 0.27 0.33 0.16

5^th^ quantile 0.62^*^ 0.38 0.41 -0.65^*^ 0.54 0.29 0.31 0.29

Thermicity **O-S Aligned Aligned** n/a n/a n/a n/a O-S

95^th^ quantile -0.50^**^ 0.82^***^ 0.87^**^ 0.15 -0.10 0.31 0.24 0.27^*^

90^th^ quantile -0.46^**^ 0.78^**^ 0.89^***^ -0.25 0.08 0.30 0.15 0.18

55^th^ quantile -0.04 0.63^***^ 0.69^***^ -0.40^*^ -0.06 0.45^*^ 0.44^*^ 0.13

50^th^ quantile -0.01 0.58^***^ 0.64^***^ -0.45^**^ -0.05 0.44^*^ 0.48^*^ 0.13

10^th^ quantile 0.04 0.45^***^ 0.64^***^ 0.00 0.11 0.26 0.29 -0.20

5^th^ quantile 0.03 0.61^***^ 0.60^***^ -0.02 -0.15 0.27 0.26 -0.22

Trait and ENVIREM data used in the quantile regression analysis were transformed to have a mean = 0 and standard deviation of 1 in order to compare slope estimates. Bold letters reflect obvious response patterns (as in Stahl et al. 2014) where upper quantiles (90th and 95^th^) and/or lower quantiles (5^th^ and 10^th^) have slopes that are significantly different from zero. DBH = diameter at breast height, SLA = specific leaf area, Growth = annual growth diameter. O-S = one-sided response pattern. * p < 0.05; ** p < 0.01; *** p < 0.001.

**Table S4**. Intercept and slope parameter estimates of significant quantile regressions for field trait-climate range models.

DBH Leaf Area Leaf Mass SLA

ENVIREM *β0 β1 β0 β1 β0 β1 β0 β1*

Clim. moist. index

95^th^ quantile

90^th^ quantile -0.24 -0.03 -0.22 -4.65 -0.74 0.003

55^th^ quantile -0.33 -0.03 -0.33 -4.69

50^th^ quantile -0.33 -0.04 -0.34 -4.50

10^th^ quantile -0.90 0.01 -0.96 0.002

5^th^ quantile -0.98 0.002

Aridity

95^th^ quantile 60.98 310.73 93.44 -0.12

90^th^ quantile 60.95 254.94 90.31 -0.11 55^th^ quantile 60.05 147.23

50^th^ quantile 58.77 163.79 81.31 -0.10

10^th^ quantile 52.79 1.18 47.37 242.37

5^th^ quantile 43.75 1.85 45.86 220.86

Annual PET

95^th^ quantile 1555.60 -10.14 819.21 67.51 896.52 8712.70 1917.64 -4.08

90^th^ quantile 1444.15 -7.64 949.11 40.67 916.16 6401.09 1878.50 -4.01

55^th^ quantile 874.70 30.53 875.14 4777.18

50^th^ quantile 880.35 26.77 885.05 3733.88

10^th^ quantile 774.95 23.85 761.00 3534.26

5^th^ quantile 679.79 32.95 759.32 3559.21

Continentality

95^th^ quantile 29.96 -0.03

90^th^ quantile 28.89 -0.02

55^th^ quantile 22.44 38.05

50^th^ quantile

10^th^ quantile 16.91 0.17

5^th^ quantile 17.22 0.14

(continued on next page)

DBH Leaf Area Leaf Mass SLA

ENVIREM *β*_0_ *β*_1_ *β0 β1 β0 β1 β0 β1*

Grow deg. days 0°C

95^th^ quantile 66177.80 -855.13 11342.94 4494.28 4792.54 705224.29

90^th^ quantile 65274.62 -834.63 4358.53 4660.99 6682.30 599586.49

55^th^ quantile 7452.24 2472.59 8217.22 330134.45 44563.99 -140

50^th^ quantile 7967.52 2256.19 8371.71 313873.06 39454.87 -118

10^th^ quantile 5998.42 1150.83 5041.55 179937.06

5^th^ quantile 4484.27 1299.69 4082.02 191297.71

Grow deg, days 5°C

95^th^ quantile 46534.4 -581.14 981.52 4393.99 3985.27 490453.80

90^th^ quantile 2873.42 3133.35 5934.02 381518.49

55^th^ quantile 6134.61 1704.16 7471.69 381518.49 32330.91 -99

50^th^ quantile 6307.28 1572.68 5312.12 238281.71 29934.92 -89

10^th^ quantile 4585.27 959.32 2871.34 164151.79

5^th^ quantile 1278.60 1198.83 2771.28 163294.55

Max. temp. coldest

95^th^ quantile 158.49 -3.14 -1.08 11.45 6.95 1734.13 210.19 -0.81

90^th^ quantile 145.13 -2.91 0.43 10.44 7.32 1286.27 217.55 -0.88

55^th^ quantile

50^th^ quantile

10^th^ quantile 62.07 6.55 -62.79 868.69 -89.75 0.41

5^th^ quantile -57.88 4.07 -75.00 1000.00 -90.54 0.41

Month by temp. 10°C

95^th^ quantile 7.75 -0.06 3.34 0.38 3.32 48.99

90^th^ quantile 6.86 -0.04 3.02 0.41 3.36 46.59

55^th^ quantile 3.13 0.23 3.32 28.86 6.50 -0.01

50^th^ quantile 2.74 0.28 2.78 35.43 6.48 -0.01

10^th^ quantile 2.24 0.18 1.52 36.91

5^th^ quantile 1.18 0.27 0.85 46.62

(continued on next page)

DBH Leaf Area Leaf Mass SLA

ENVIREM *β*_0_ *β*_1_ *β*_0_ *β*_1_ *β*_0_ *β*_1_ *β*_0_ *β*_1_

Min. temp. warmest

95^th^ quantile 37.93 13.55 85.04 1021.86

90^th^ quantile 51.72 10.70 43.34 1675.68

55^th^ quantile 32.40 9.63 26.48 1328.14 157.78 -0.45

50^th^ quantile 23.57 10.35 20.10 1396.74 143.74 -0.38

10^th^ quantile 11.62 6.73 3.20 1199.99

5^th^ quantile -5.24 8.28 2.18 1207.46

PET driest quarter

95^th^ quantile 120.77 6.16 116.01 958.32 273.64 -0.71

90^th^ quantile 122.24 5.55 118.28 848.36 252.94 -0.61

55^th^ quantile 99.69 6.06 106.85 692.33

50^th^ quantile 96.92 5.78 101.65 750.91

10^th^ quantile

5^th^ quantile

PET coldest quarter

95^th^ quantile 63.49 -0.85 18.45 3.19 18.82 536.59 81.71 -0.25

90^th^ quantile 56.61 -0.73 19.26 2.65 21.31 332.60 77.13 -0.24

55^th^ quantile 35.67 -0.37

50^th^ quantile 33.90 -0.34

10^th^ quantile 7.43 1.27 7.12 169.88 2.08 0.08

5^th^ quantile 8.11 118.92 1.23 0.09

PET warmest quarter

95^th^ quantile 205.27 -0.54 152.79 651.40

90^th^ quantile 201.27 -0.53 147.79 741.67 226.83 -0.29

55^th^ quantile 141.93 4.02 139.30 614.95 208.44 -0.25

50^th^ quantile 142.33 3.86 139.81 590.12 201.16 -0.22

10^th^ quantile 130.55 3.53 133.73 492.44 178.17 -0.19

5^th^ quantile 126.79 567.14 169.77 -0.15

(continued on next page)

DBH Leaf Area Leaf Mass SLA

ENVIREM *β*_0_ *β*_1_ *β*_0_ *β*_1_ *β*_0_ *β*_1_ *β*_0_ *β*_1_

PET wettest quarter

95^th^ quantile 118.89 6.60 129.69 655.88 203.91 -0.28

90^th^ quantile 134.47 388.52

55^th^ quantile 69.20 0.36

50^th^ quantile 65.65 0.37

10^th^ quantile -16.35 7.80 -27.00 1301.19 -138.33 1.20

5^th^ quantile -24.42 7.39 -30.53 1271.04

PET seasonality

95^th^ quantile 5454.15 150.60 5333.61 21530.33 7708.36 -9.27

90^th^ quantile 5423.60 143.60 5406.38 19201.71 7700.64 -9.23

55^th^ quantile 5368.57 88.56 5136.06 16645.18 7103.27 -8.22

50^th^ quantile 5371.88 81.84 5158.22 15037.17 6915.84 -7.44

10^th^ quantile 4721.45 23.89 4888.93 73.85 4874.17 13220.45 6945.03 -9.88

5^th^ quantile 4513.10 28.94 6747.90 -8.97

Thermicity

95^th^ quantile 268.09 -4.87 -83.70 4201.07

90^th^ quantile 248.38 -4.42 -109.73 4320.49

55^th^ quantile -142.38 3329.41 179.02 -1.15

50^th^ quantile -145.07 22.86 -140.16 3096.12 194.92 -1.28

10^th^ quantile -192.27 17.91 -216.54 3111.61

5^th^ quantile -257.25 24.20 -213.56 2910.20

*β*_0_ = intercept, *β*_1_ = slope. DBH = diameter at breast height, SLA = specific leaf area.

**Table S5**. Intercept and slope parameter estimates of significant quantile regressions for common garden trait-climate range models.

Growth Leaf Area Leaf Mass SLA

ENVIREM *β*_0_ *β*_1_ *β*_0_ *β*_1_ *β*_0_ *β*_1_ *β*_0_ *β*_1_

Clim. moist. index

95^th^ quantile

90^th^ quantile

55^th^ quantile -0.21 -0.08 -0.30 -6.84 -1.27 0.01

50^th^ quantile -0.34 -0.06 -0.35 -5.98 -1.35 0.01

10^th^ quantile

5^th^ quantile

Aridity

95^th^ quantile 64.54 3.14 66.84 289.67

90^th^ quantile 63.80 274.57

55^th^ quantile

50^th^ quantile

10^th^ quantile

5^th^ quantile

Annual PET

95^th^ quantile

90^th^ quantile 2075.81 -484.97

55^th^ quantile 814.39 58.60 864.34 4945.08

50^th^ quantile 817.92 55.86 834.00 5286.17

10^th^ quantile 636.17 68.57 676.13 6291.04 1599.10 -6.26

5^th^ quantile 1738.22 -7.61

Continentality

95^th^ quantile

90^th^ quantile

55^th^ quantile

50^th^ quantile

10^th^ quantile 7.58 8.39

5^th^ quantile 5.89 9.21

(continued on next page)

Growth Leaf Area Leaf Mass SLA

ENVIREM *β*_0_ *β*_1_ *β*_0_ *β*_1_ *β*_0_ *β*_1_ *β*_0_ *β*_1_

Grow deg. days 0°C

95^th^ quantile 127732.73 -49769.59 -1762.86 11300.58 -36707.95 713.90

90^th^ quantile -28838.96 626.11

55^th^ quantile 7804.14 3865.74 9010.84 356955.35

50^th^ quantile 1270.40 4951.95 7852.48 365462.33

10^th^ quantile

5^th^ quantile

Grow deg, days 5°C

95^th^ quantile -35856.32 619.08

90^th^ quantile

55^th^ quantile 4488.30 3080.71 38612.66 -136723.91

50^th^ quantile 3467.16 3247.70 20203.99 85055.87

10^th^ quantile

5^th^ quantile

Max. temp. coldest

95^th^ quantile 388.28 -190.08

90^th^ quantile 349.09 -171.03

55^th^ quantile 120.30 -59.83

50^th^ quantile -46.67 13.35

10^th^ quantile -87.16 16.33 -88.40 1852.92 238.28 -2.30

5^th^ quantile -74.87 1355.79 211.35 -2.15

Month by temp. 10°C

95^th^ quantile 1.85 0.04

90^th^ quantile 2.94 0.70 1.11 0.04

55^th^ quantile 2.27 0.57

50^th^ quantile 2.29 0.55 2.48 50.61

10^th^ quantile 1.70 44.49

5^th^ quantile 0.30 62.33

(continued on next page)

Growth Leaf Area Leaf Mass SLA

ENVIREM *β*_0_ *β*_1_ *β*_0_ *β*_1_ *β*_0_ *β*_1_ *β*_0_ *β*_1_

Min. temp. warmest

95^th^ quantile 87.61 11.66 86.10 1353.77

90^th^ quantile 72.88 12.94

55^th^ quantile 22.20 16.94

50^th^ quantile 23.88 15.63 33.52 1318.88

10^th^ quantile 6.69 1083.04

5^th^ quantile -0.36 1189.68

PET driest quarter

95^th^ quantile 12.22 1.41

90^th^ quantile

55^th^ quantile

50^th^ quantile

10^th^ quantile

5^th^ quantile

PET coldest quarter

95^th^ quantile 118.14 -48.26

90^th^ quantile 111.81 -45.19

55^th^ quantile

50^th^ quantile 7.38 3.71

10^th^ quantile 3.71 2.90 5.69 252.90

5^th^ quantile 6.41 1.85 5.50 251.56 54.46 -0.39

PET warmest quarter

95^th^ quantile

90^th^ quantile

55^th^ quantile 143.27 6.42

50^th^ quantile 130.70 8.72

10^th^ quantile

5^th^ quantile

(continued on next page)

Growth Leaf Area Leaf Mass SLA

ENVIREM *β*_0_ *β*_1_ *β*_0_ *β*_1_ *β*_0_ *β*_1_ *β*_0_ *β*_1_

PET wettest quarter

95^th^ quantile

90^th^ quantile 248.98 -57.73

55^th^ quantile 302.70 -107.36 331.02 -1.87

50^th^ quantile 265.69 -89.38 366.02 -1.87

10^th^ quantile -55.84 22.59 -75.95 3263.58 512.89 -4.16

5^th^ quantile -45.37 17.69 -39.15 1910.44 537.44 -4.42

PET seasonality

95^th^ quantile 6060.62 13596.03

90^th^ quantile

55^th^ quantile

50^th^ quantile

10^th^ quantile

5^th^ quantile

Thermicity

95^th^ quantile -174.93 2.95

90^th^ quantile

55^th^ quantile -166.22 43.11 -141.06 3915.88

50^th^ quantile -164.82 42.02 -155.02 4194.41

10^th^ quantile

5^th^ quantile

*β*_0_ = intercept, *β*_1_ = slope. Growth = annual growth diameter, SLA = specific leaf area.

**Figure S1**. Trait variation across elevation gradients.

**Figure S2**. Trait-climate range relationships using principle component analysis.

**Figure S3**. Plant size and growth trait-climate range quantile regressions.

**Figure S4**. Leaf area-climate range quantile regressions.

**Figure S5**. Leaf mass-climate range quantile regressions.

**Figure S6**. Specific leaf area-climate range quantile regressions.
